# Ce/Sm/Sr-Incorporating Ceramic Scaffolds Obtained via Sol-Gel Route

**DOI:** 10.3390/ma14061532

**Published:** 2021-03-21

**Authors:** Sorin-Ion Jinga, Ana-Maria Anghel, Silvia-Florena Brincoveanu, Raluca-Maria Bucur, Andrei-Dan Florea, Bianca-Irina Saftau, Stefania-Cristina Stroe, Andreea-Ioana Zamfirescu, Cristina Busuioc

**Affiliations:** 1 Faculty of Applied Chemistry and Materials Science, University POLITEHNICA of Bucharest, RO-011061 Bucharest, Romania; sorinionjinga@yahoo.com; 2 Faculty of Medical Engineering, University POLITEHNICA of Bucharest, RO-011061 Bucharest, Romania; ana.maria_anghel@yahoo.com (A.-M.A.); brincoveanu.silvia@gmail.com (S.-F.B.); ralucabucur26@yahoo.com (R.-M.B.); andreidan96@yahoo.com (A.-D.F.); irina.saftau@yahoo.ro (B.-I.S.); stefi_1196@yahoo.com (S.-C.S.); zamfirescu.andreea96@gmail.com (A.-I.Z.)

**Keywords:** ceramics, scaffolds, rare earths, Sol-gel, compression strength, bone substitutes

## Abstract

Three different inorganic scaffolds were obtained starting from the oxide system SiO_2_‒P_2_O_5_‒CaO‒MgO, to which Ce^4+^/Sm^3+^/Sr^2+^ cations were added in order to propose novel materials with potential application in the field of hard tissue engineering. Knowing the beneficial effects of each element, improved features in terms of mechanical properties, antibacterial activity and cellular response are expected. The compositions were processed in the form of scaffolds by a common sol-gel method, followed by a thermal treatment at 1000 and 1200 °C. The obtained samples were characterized from thermal, compositional, morphological and mechanical point of view. It was shown that each supplementary component triggers the modification of the crystalline phase composition, as well as microstructural details. Moreover, the shrinkage behavior is well correlated with the attained compression strength values. Sm was proven to be the best choice, since in addition to a superior mechanical resistance, a clear beneficial influence on the viability of 3T3 fibroblast cell line was observed.

## 1. Introduction

Ceramic scaffolds with optimized properties in terms of oxide composition, crystallinity degree, porosity arrangement and mechanical characteristics were proposed in the last decades as suitable implantable materials [1,2]. Moreover, if their impact on various types of cells, rate of degradation in the physiological medium or bonding capability towards natural tissues can be induced or even controlled, most of the requirements imposed by the practice are meet. The current challenge is to take a step forward towards reaching improved or even smart devices for medical use by integrating such materials in commercial products. To make this possible, new systems were investigated and unexpected responses were acquired for elements [3] or compounds [4,5] that were previously declared remarkable for other applications than the biological ones. Thus, a wide range of rare earths were integrated in bioceramics, the results being most of the time appropriate for developing tunable substitutes or personalized therapies [6,7]. As well, the strategy of releasing elements with therapeutic effects from the implantable biomaterials in order to efficiently and locally treat certain diseases represents a modern approach of bioengineering and regenerative medicine [8].

Strontium (Sr) is one of the most approached alkaline earth elements for biologically triggering superior integration of artificial tissue substituting bodies. First, it was employed as dopant for hydroxyapatite, replacing Ca in the crystalline network and inducing local distortions in the host lattice, additionally enhancing the dissolution rate or providing antibacterial action [9,10]. It was also integrated in different glass formulations due to its positive impacts on the mechanical strength, drug release and osteogenesis; the biological improvements were substantial both in vitro and in vivo, even at very small concentrations (below 0.2 wt.% Sr) [8,11]. In the case of Sr-containing glass ceramics, in vitro bioactivity, high antibacterial and antifungal action, as well as excellent osseointegration during short term implantation in animal models were found; 5 wt.% SrO seems to be the optimal content [12,13]. The antimicrobial properties can be further enhanced by appealing to different combinations [14,15]. Other authors [16] demonstrated the efficiency of Sr incorporation in osteoporotic bone regeneration. Overall, its favorable influences led to the production of materials belonging to different compositional or dimensional classes, including fibers [17], thin films [18] and composites [19].

Cerium (Ce) has received much attention due to its interesting properties, generated by the convenient switch between oxidation states (Ce^4+^, Ce^3+^); recently, it was revealed to have multi-enzyme-mimetic properties that make it attractive for bioapplications [20]. When dopant for hydroxyapatite, Ce^3+^ ions ensure sufficient mechanical strength for use in the replacement of spongy bone [21]. CeO_2_ nanoparticles employed as decoration for cancellous bone grafts significantly promoted the blood vessel development process [22]. Bioactive glassy coatings containing CeO_2_ nanocrystals (5 mol% CeO_2_) were proposed as suitable supports for fibroblast cells development, hindering in the same time bacteria colonization [23]. *3*D-printed composite scaffolds based on CaSiO_3_ and maximum 15 mol% CeO_2_ showed enhanced osteogenic activity [24], while polymeric scaffolds loaded with CeO_2_ nanoparticles organized into self-assembled line patterns enabled aligned cell growth [25]. The incorporation of CeO_2_ nanostructures into composite polymeric scaffolds increased the mechanical properties and biomineralization, enhanced the cellular proliferation, assisted the osteogenic differentiation and showed very good free radical-scavenging capabilities [26].

Unlike antibiotics, CeO_2_ nanoparticles represent important antibacterial agents due to their relatively low toxicity to normal cells and their distinct functioning mechanism, based on the reversible conversion between Ce^3+^ and Ce^4+^ valence states, which endows this oxide with both anti- and pro-oxidative properties. Zhang et al. [27] discussed the underlying mechanism, concluding that the initial and key step is the electrostatic interaction at the bacterial membrane level, followed by the generation of reactive oxygen species and physical damage of microorganisms. The corresponding nanostructures are also powerful self-regenerative antioxidant materials, that behave as strong reactive oxygen species scavengers; the tests performed on neuronal cells demonstrated strong antioxidant properties and beneficial effects on neurite development and alignment [28]. Likewise, it was reported that CeO_2_ nanoparticles play a key role in the enhancement of angiogenesis, but care should be taken when using higher concentrations (more than 3 wt.% CeO_2_) because they may induce local inflammatory reactions [29].

Samarium (Sm) is a rarer choice when it comes to biological applications. It was tested in the form of oxide as bone substituting material and the results revealed that the corresponding surfaces provide appropriate supports for osteoblast-like cells adhesion, proliferation, differentiation and mineralization at short terms [30]. When used as dopant for hydroxyapatite, it had a beneficial effect on cell viability and ensured antibacterial activity [31]. The glasses with Sm content (maximum 2 mol% Sm_2_O_3_) showed improved osteoblastic behavior and antibacterial effects, dependent on the amount of Sm in the composition [32]. Ershad et al. [33] studied melt-derived bioactive glasses with up to 4 wt.% Sm_2_O_3_; they found that nucleation and crystal growth temperature decrease, while bioactivity and mechanical properties improve with Sm_2_O_3_ concentration increasing. Sm-doped thin films were prepared from the desire to ensure two key features contributing to a better outcome following bone graft implantation: rapid osseointegration and infection avoidance; the tests evidenced antifungal properties, simultaneously with lack of toxicity against regular cell lines for Sm contents below 1 mol% [34,35]. Moreover, ^153^Sm isotope was demonstrated to be an efficient radiotracer [36] and helpful agent for nuclear therapy through beta emission [37,38]. It has also been reported that the amount of adding multiple rare earth oxides can dramatically influence the bioactivity of bioceramic coatings [39].

In this work, Ce and Sm-containing glass compositions were proposed and prepared for the first time; the repercussions of rare earth cations on the physicochemical and biological properties were then evaluated. Moreover, an alkaline earth element (Sr) was also integrated in the same matrix, serving as comparison counterpart. All fabricated scaffolds were investigated from multiple perspectives (compositional, morphological, mechanical and biological), with the aim of showing particular improvements achieved through such additions (Ce, Sm or Sr).

## 2. Experimental

The materials size, texture and surface chemistry can be adjusted through the approached wet-chemistry method, in order to propose more biologically active systems. The sol-gel-derived samples exhibit, besides the well-known purity and homogeneity [40], a higher potential in terms of ion release rate, namely tailored features, in agreement with the clinical needs [8].

As a consequence, the precursor powders were synthesized through a common sol-gel route. The employed reagents were as follows: tetraethyl orthosilicate (Si(OC_2_H_5_)_4_, TEOS, 98%, Aldrich, St. Louis, MO, USA), triethyl phosphate (PO(OC_2_H_5_)_3_, TEP, ≥99%, Merck, St. Louis, MO, USA), calcium nitrate tetrahydrate (Ca(NO_3_)_2_·4H_2_O, 99%, Merck, St. Louis, MO, USA), magnesium nitrate hexahydrate (Mg(NO_3_)_2_·6H_2_O, 99%, Merck, St. Louis, MO, USA), ammonium cerium(IV) nitrate ((NH_4_)_2_Ce(NO_3_)_6_, ≥98%, Sigma-Aldrich, St. Louis, MO, USA), samarium nitrate hexahydrate (Sm(NO_3_)_3_·6H_2_O, 99.9%, Aldrich, St. Louis, MO, USA) and strontium nitrate (Sr(NO_3_)_2_, ≥99%, Sigma-Aldrich, St. Louis, MO, USA). The selected oxide compositions are shown in Table 1. For each one, the alkoxides were hydrolysed, while the nitrates were dissolved, leading, after mixing and homogenization for 1 h under magnetic stirring at room temperature, to one final clear solution. The latter was converted into gel after 12 h, which was then aged for 24 h and dried at 80 °C for 12 h. All three precursor gels were calcined at 600 °C for 2 h. In order to acquire green bodies, the calcined powders were granulated with a 2 wt.% aqueous solution of polyvinyl alcohol, uniaxially pressed at 220 MPa as cylinders (13 mm diameter and 6 mm height) and finally sintered at 1000 °C (Ce-1000, Sm-1000, Sr-1000) or 1200 °C (Ce-1200, Sm-1200, Sr-1200) for 2 h under static air.

The samples were investigated from the stage of dried gel to the one of sintered ceramic by employing the following techniques: thermal analysis (Shimadzu DTG-60 equipment, *temperature* = 25–1000 °C, *heating rate* = 10 °C/min, static air), X-ray diffraction (XRD, Shimadzu XRD 6000 diffractometer, Ni-filtered Cu Kα radiation, λ = 1.54 Å, 2θ = 20–60 °, *scan speed* = 2°/min), Fourier-transform infrared spectroscopy (FTIR, Thermo Scientific Nicolet iS50 spectrophotometer, *wavenumber* = 400–2000 cm^−1^), scanning electron microscopy coupled with energy-dispersive X-ray spectroscopy (SEM+EDS, FEI Quanta Inspect F electron microscope, samples coated with a thin layer of gold by DC magnetron sputtering), compression strength measurements (Instron 3382 universal testing system, circular cross-section, *diameter* = 13 mm, *speed* = 1 mm/min).

The preliminary biological response was quantitatively assessed through the viability/cytotoxicity test using the LIVE/DEAD kit for mammalian cells, that allows fluorescent marking of living cells with Calcein-AM and dead cell nucleus with Ethidium Homodimer-1 [41]. The evaluation was performed by confocal microscopy (Carl Zeiss LSM 880 confocal microscope) after 7 days in 3T3 fibroblast cell culture.

## 3. Results and Discussion

**Figure 1:**
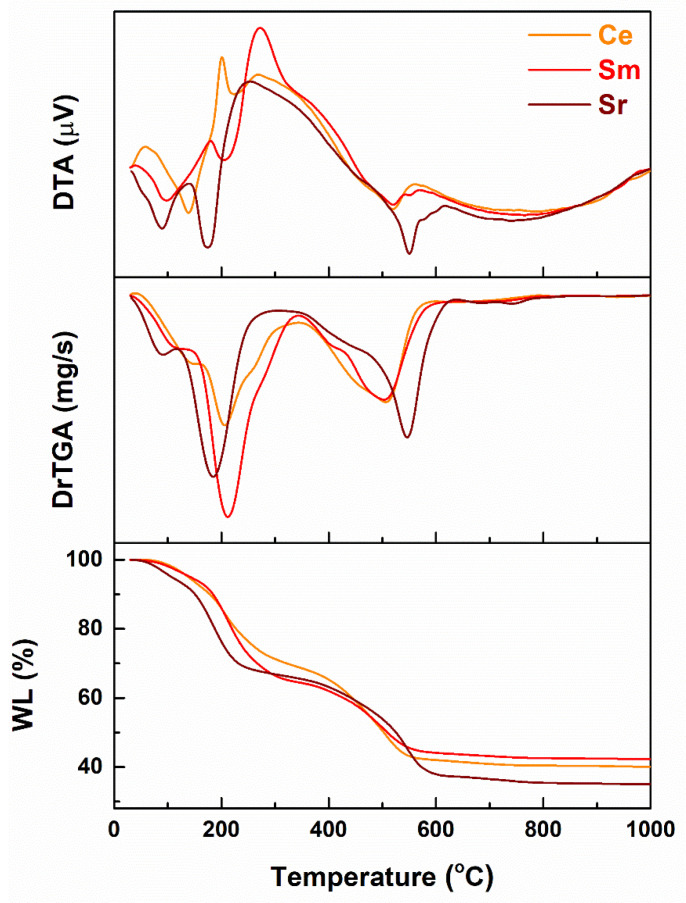
Thermal analyses of the dried gels (*WL* = weight loss, *DrTGA* = derivative of the thermogravimetric analysis with respect to time, *DTA* = differential thermal analysis).

All three dried gels were subjected to thermal analysis in order to reveal potential differences triggered by the incorporation of an alkaline earth metal or two lanthanides. As it can be seen in Figure 1, the curves are only slightly modified from one case to another, namely small shifts in temperature or variations in intensity. However, the major weight loss steps and thermal pattern are preserved. Three main weight loss stages can be observed in the investigated temperature range, as follows: the first one from room temperature to about 150 °C, the second one extends to around 325 °C, while the third one ends around 600 °C; these corresponds to total values of 60% for Ce, 58% for Sm- and 65% for Sr-containing gels. It can be stated that the identified weight losses are correlated with endothermic processes of residual solvents evaporation at low temperatures, followed by an endothermic process corresponding to organic burn out and then an endothermic one associated with nitrate groups decomposition. At a closer look, it is visible that Ce and Sm-including gels have very similar behaviors, which was expected, since they belong to the same family of elements. Concluding, the weight loss above 600 °C is extremely low (below 3%), fact that led to the selection of this value as calcination temperature.

**Figure 2:**
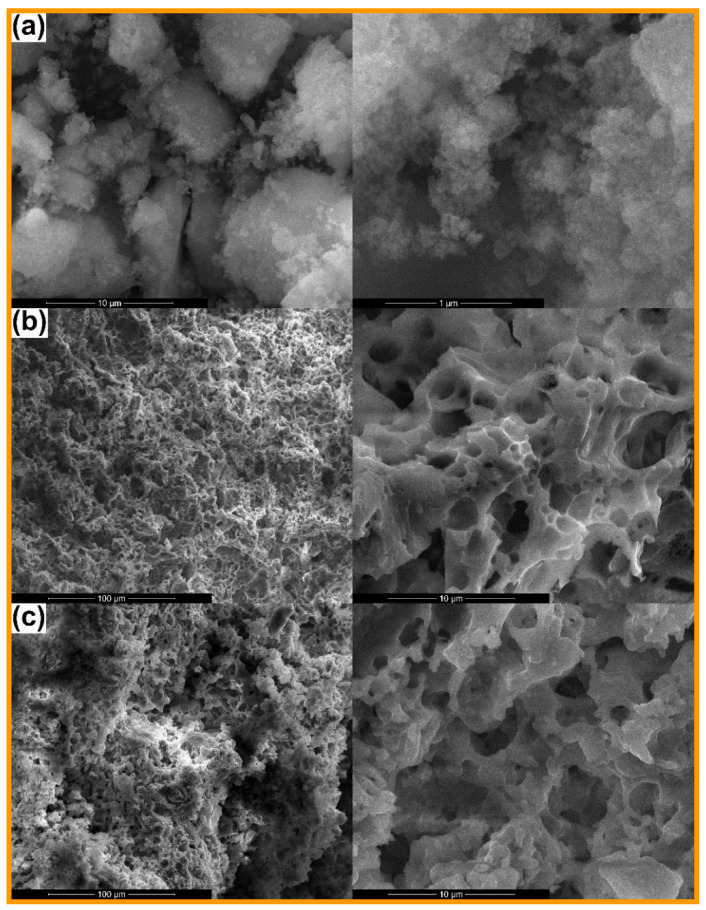
SEM images of: (**a**) Ce calcined powder (10,000× and 100,000×), (**b**) Ce-1000 sintered scaffold (1000× and 10,000×) and (**c**) Ce-1200 sintered scaffold (1000× and 10,000×).

**Figure 3:**
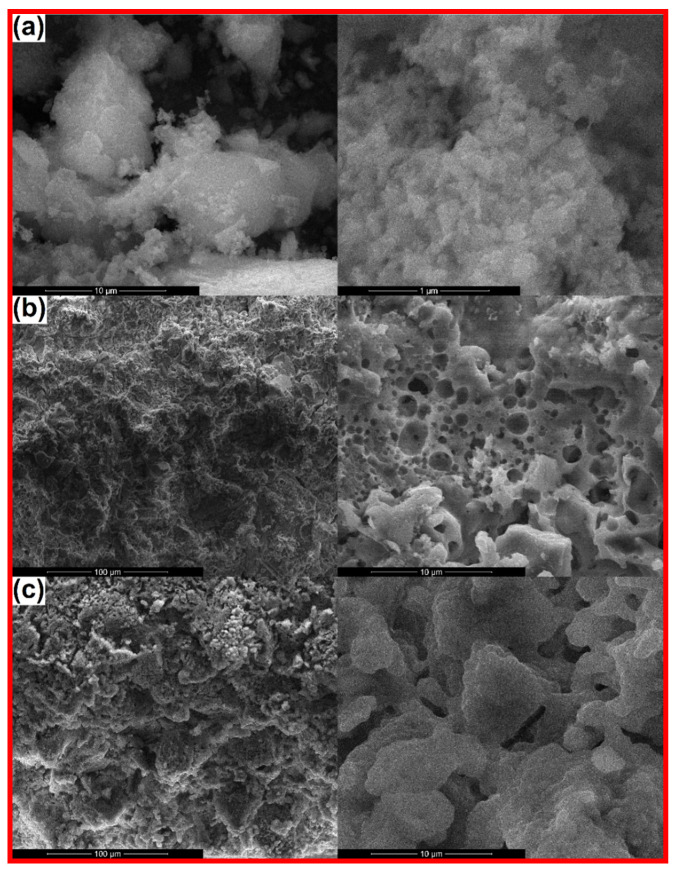
SEM images of: (**a**) Sm calcined powder (10,000× and 100,000×), (**b**) Sm-1000 sintered scaffold (1000× and 10,000×) and (**c**) Sm-1200 sintered scaffold (1000× and 10,000×).

**Figure 4:**
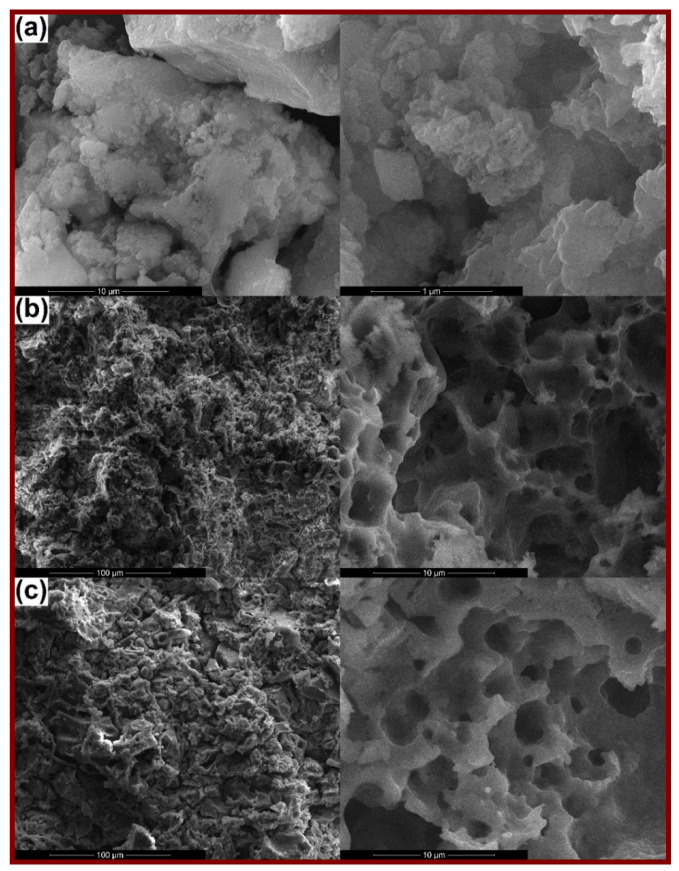
SEM images of: (**a**) Sr calcined powder (10,000× and 100,000×), (**b**) Sr-1000 sintered scaffold (1000× and 10,000×) and (**c**) Sr-1200 sintered scaffold (1000× and 10,000×).

The morphology of the calcined powders is presented in Figure 2a, Figure 3a and Figure 4a, together with the microstructure of the scaffolds sintered at 1000 °C (Figure 2b, Figure 3b and Figure 4b) and 1200 °C (Figure 2c, Figure 3c and Figure 4c). Analyzing comparatively the available SEM images, the powder finesse varies from Ce-incorporating composition (particles below 20 nm gathered in fluffy agglomerates; Figure 2a) to Sm-containing one (entities below 100 nm connected in much dense aggregates, probably composed of smaller particles that are tightly packed; Figure 3a) and finally Sr-including one (particles intimately linked in massive blocks, in which the borders are cancelled; Figure 4a). These findings are well correlated with the crystalline characteristics evidenced by X-ray diffraction (Figure 7). Thus, Ce-containing powder is the only one that individualizes as a distinct ordered phase (CeO_2_) from the stage of powder, this probably hindering the particle growth. After applying the high temperature thermal treatment, the powders convert into three-dimensional bodies with a well-defined network, containing pores or crossed by channels. The formed walls and their connections ensure the mechanical resistance of the entire ensemble. Obviously, there is a porosity reduction when the sintering temperature increases from 1000 to 1200 °C, due to densification associated with material diffusion. Regarding Ce-including composition, the low temperature scaffold is defined by the existence of spherical pores with sizes ranging from less than 100 nm to 4 μm, distributed sometimes in a spongy appearance (Figure 2b), while the high temperature one seems more densified (Figure 2c), based on porosity decrease through the disappearance of small pores.

When it comes to Sm-incorporating composition, the scaffold looks like a foam at 1000 °C, with pores belonging to a narrower dimensional range (maximum 2 μm) and a more homogenous aspect on large areas (Figure 3b). This could be explained based on the action of Sm as dopant, favoring the material densification. At 1200 °C, the situation is completely changed, in the sense that large blocks (exceeding 5 μm) emerge (Figure 3c), probably associated with the modification of mineralogical composition, basically the disappearance of Ca_2_Sm_8_(SiO_4_)_6_O_2_ crystalline compound when going from 1000 to 1200 °C (Figure 7). Indeed, porosity is still present between these massive building units, but the mechanical characterization proved that it does not imply a detrimental influence in this direction (Figure 8).

Finally, for Sr-containing composition, the same porous aspect is observed at 1000 °C, with pores in a broad dimensional range (Figure 4b), while the increase of the thermal treatment temperature to 1200 °C allows the achievement of a continuous matrix pierced by a limited number of elongated pores or branched channels (Figure 4c), as a result of primary pores coalescence during sintering, process that does not seem to be accompanied by a significant densification; this last statement is sustained by the close values obtained for the shrinkage parameter (0.13% for 1000 °C versus 1.93% for 1200 °C), showing that just a porosity rearrangement occurred (Figure 8).

Summarizing, all scaffolds contain a certain degree of porosity, with significant differences in terms of pores size, interconnectivity and distribution. The most densified scaffolds are the ones containing Sm, whereas the airiest are encountered in the case of Ce addition. These findings will pronouncedly affect the response of the bulks under mechanical loads (Figure 8).

**Figure 5:**
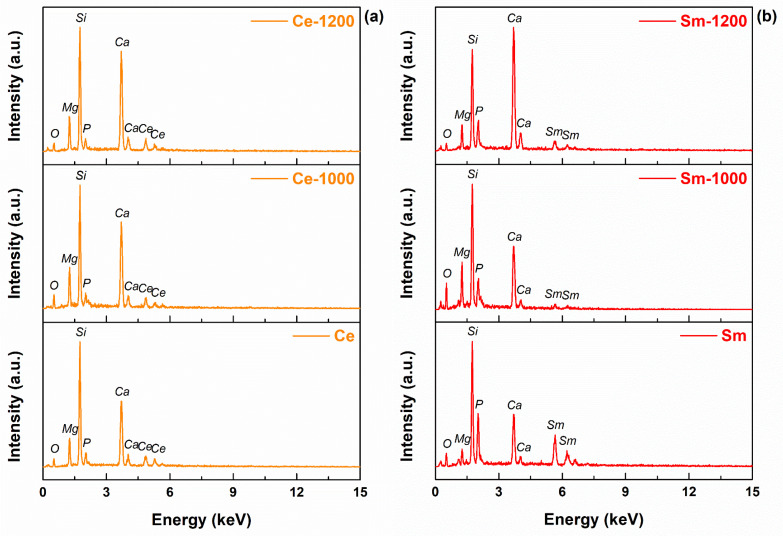

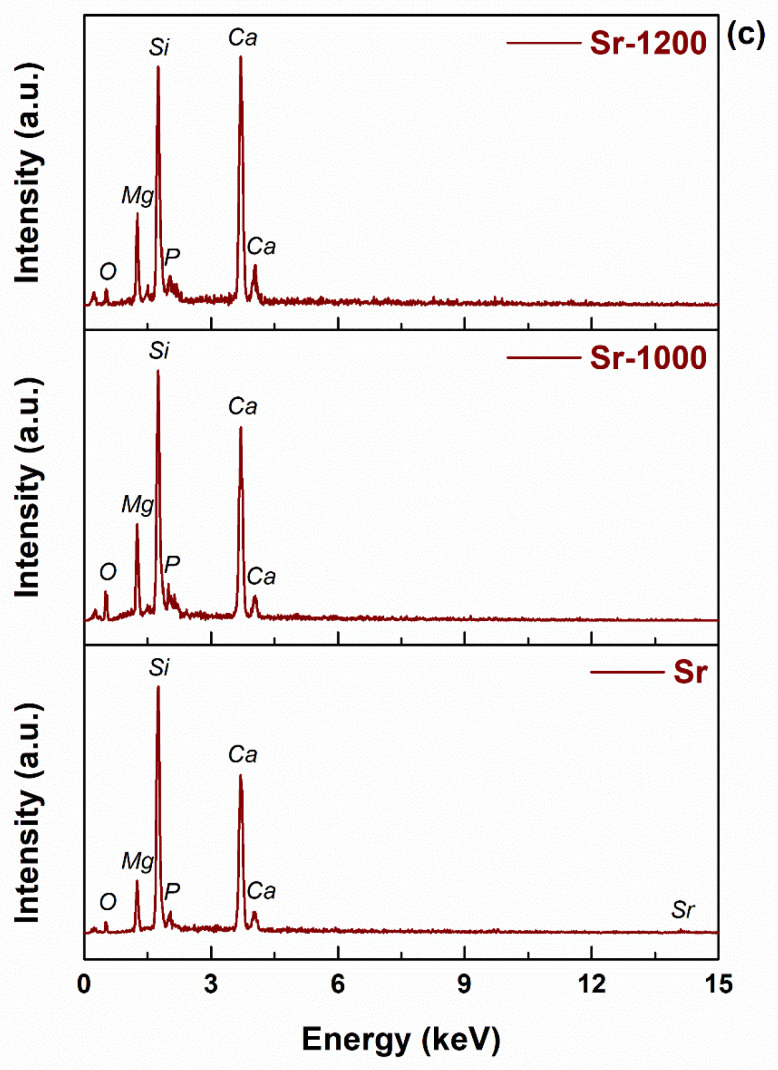
EDS spectra of the developed materials: (**a**) Ce-containing composition, (**b**) Sm-containing composition and (**c**) Sr-containing composition.

The elemental composition was also investigated, the EDS spectra being presented in Figure 5. All samples show signals coming from the matrix (Si, P, Ca, Mg, O) and incorporating elements (Ce, Sm, Sr). If Ce appears evenly in the associated materials both before and after the thermal treatments, Sm displays a higher intensity in the stage of precursor powder, fact that could be linked to the occurrence of a concentration gradient in the final three-dimensional bodies. The detection of Sr is more difficult, since the characteristic lines arise at high energies; only in the case of the calcined powder, a small peak was recorded, which confirms its maintenance in the designed composition. It is obvious that Sr is still present in the sintered scaffolds since it was detected in the precursor powder, but either it needs higher excitation energy to observe it by EDS or it could be inhomogeneously integrated as dopant in certain crystalline lattices or as rich areas in the amorphous component, this explaining its absence from the corresponding spectra if the investigated areas were by chance poor in Sr.

In order to have a better evaluation on this type of composition, the previously spectra were processed and semi-quantitative data were extracted and shown in Table 2. To get comparable information, the acceleration voltage was maintained at 30 kV, while the area of the investigated surface was kept constant by using the same magnification every time. The variations are explained based on the equipment performance (detection limit of 2–3%), most of them being acceptable. Otherwise, the values show that the targeted elements are present within the powders and scaffolds, while the peculiar differences may signal local inhomogeneities.

**Figure 6:**
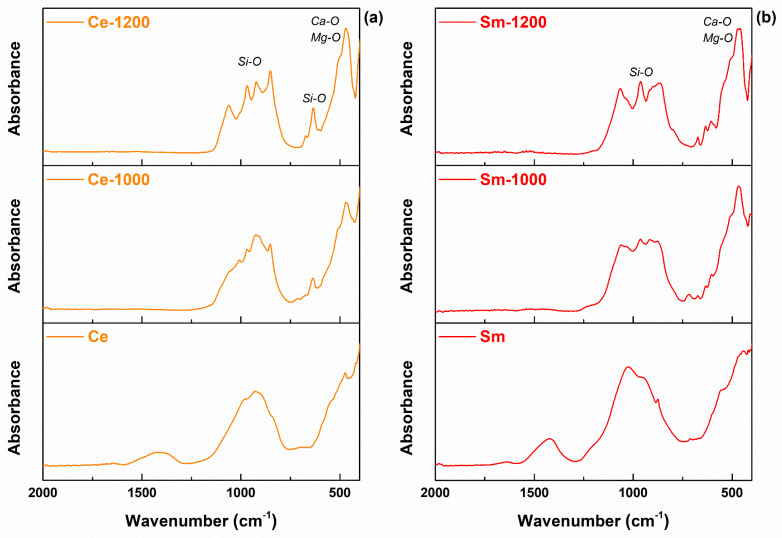

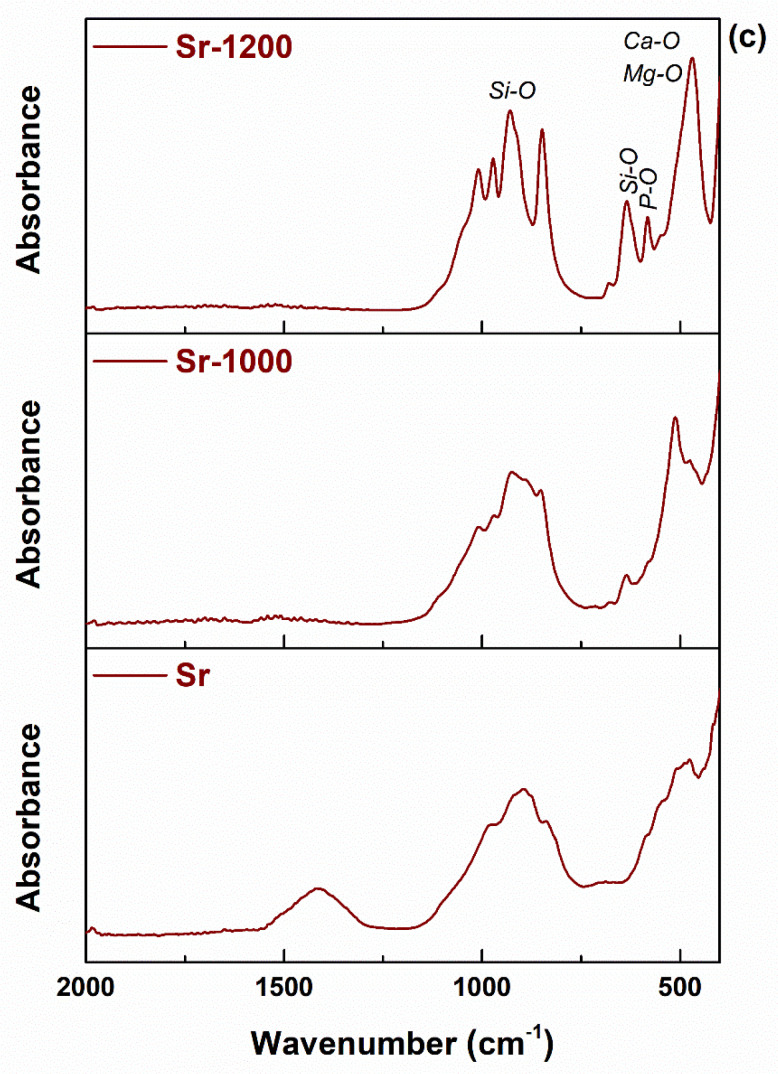
FTIR spectra of the developed materials: (**a**) Ce-containing composition, (**b**) Sm-containing composition and (**c**) Sr-containing composition.

**Figure 7:**
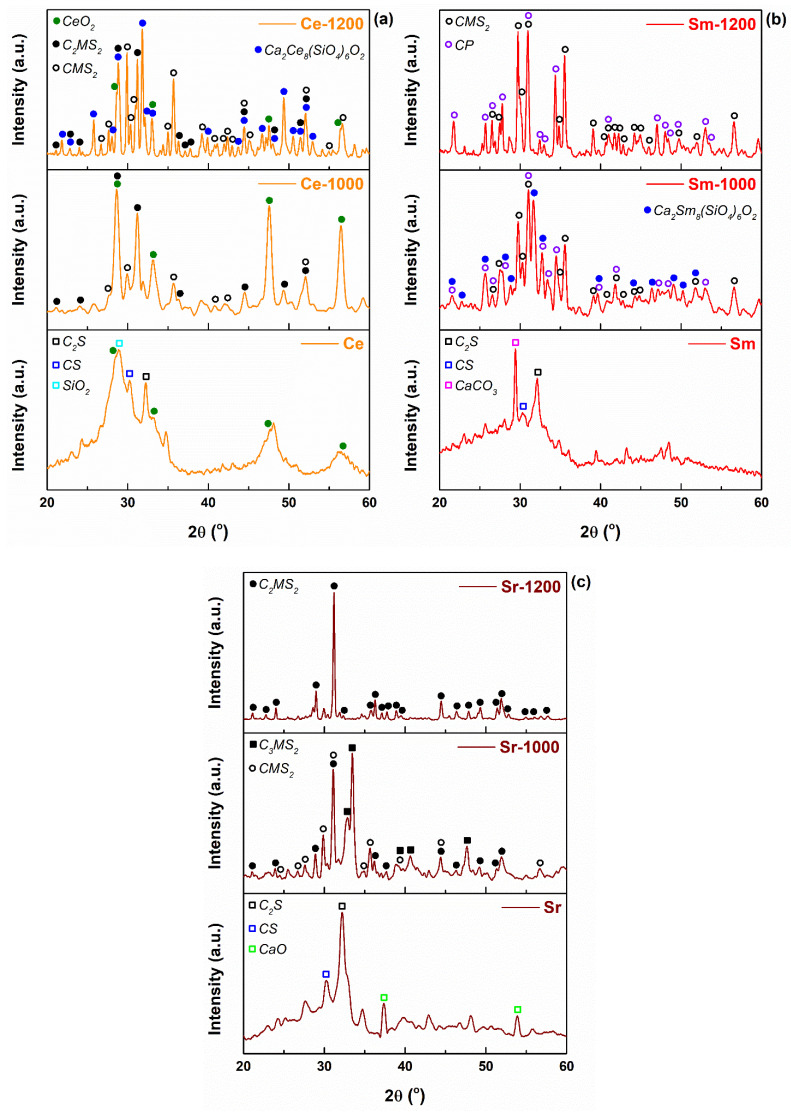
XRD patterns of the developed materials: (**a**) Ce-containing composition, (**b**) Sm-containing composition and (**c**) Sr-containing composition.

Afterwards, the chemical bonds established between the formerly identified elements were studied through the FTIR spectra displayed in Figure 6. The situation is similar for all three elements (Ce, Sm, Sr), the tendency of the recorded curves being clearly divided in two main classes: spectra large with large bands resulted from the overlapping of adjacent multiple signals (for the precursor powders and scaffolds sintered at 1000 °C) and spectra with well-defined individual maxima (for the scaffolds thermally treated at 1200 °C). This characterization technique is sensitive to the degree of ordering encountered within the samples and proves in this way the increase of crystallinity when rising the temperature to 1200 °C, especially for Si‒O bonds and [SiO_4_]^4−^ tetrahedra. Basically, the vibrational features were assigned to three main types of bonds: Si‒O, Ca‒O and Mg‒O [42], in correlation with the elemental (Figure 5) and mineralogical (Figure 7) details. Another specificity is related to the emergence of a supplementary large band in the powders spectra, namely the one centered at approximately 1420 cm^−1^, which was attributed to the presence of residual nitrates [43].

Figure 7 exhibits the XRD patterns of the obtained materials, both before and after sintering at high temperatures; this allows a precise observation of material evolution upon heating. The powders calcined at 600 °C gathered patterns that highlight the incipient crystallization through the halo placed below 35° and wide peaks shaped either over the halo or to its right; the halo is the fingerprint of the vitreous phase, while the peaks were generally attributed to two types of calcium silicates: Ca_2_SiO_4_ = C_2_S (larnite, ICDD 00-083-0465) and CaSiO_3_ = CS (wollastonite, ICDD 00-072-2297), both with monoclinic structure; however, the additional diffraction peaks were linked to the presence of primary oxides or carbonated species, as follows: monoclinic SiO_2_ (coesite, ICDD 00-083-1828) or other polymorphs, cubic CaO (ICDD 00-077-2376) or rhombohedral CaCO_3_ (ICDD 00-072-1937). The diffraction halo can be well discerned even for the scaffolds thermally treated at 1000 °C, which suggests that an appreciable quantity of glass remained within these samples. In the case of Sr-incorporating scaffolds, a mixture of two calcium magnesium silicates: Ca_3_MgSi_2_O_8_ = C_3_MS_2_ (merwinite, ICDD 00-074-0382) and CaMgSi_2_O_6_ = CMS_2_ (diopside, ICDD 00-083-1817), both with monoclinic symmetry, were observed after the thermal treatment at 1000 °C, and a single phase: tetragonal Ca_2_MgSi_2_O_7_ = C_2_MS_2_ (akermanite, ICDD 00-083-1815) after thermally treating at 1200 °C. Basically, when considering the composition defined by the majority oxides (SiO_2_, CaO, MgO), it falls within the primary crystallization field of akermanite, while the areas thermodynamically assigned to merwinite and diopside are adjacent to the border. Moreover, starting from the structure of akermanite (high temperature major compound), with tetrahedra having Si cation in the center ([SiO_4_]^4−^) and sharing a corner of O so that to form bipyramids ([Si_2_O_7_]^6−^), Mg cations tetrahedrally surrounded by O anions ([MgO_4_]^6−^) and Ca cations 8-fold coordinated ([CaO_8_]^14−^) [44], as well as the ionic radii of the involved cations (Si^4+^(IV) = 0.26 Å, Mg^2+^(IV) = 0.57 Å, Ca^2+^(VIII) = 1.12 Å), it seems that Sr cations, with an ionic radius of Sr^2+^(VIII) = 1.26 Å, can only substitute Ca cations within the ordered networks. Thus, Sr is either dopant for the mentioned calcium magnesium silicates, stressly occupying the sites of Ca [45] or remains distributed in the amorphous phase, along with P. Ce generates a separate oxide phase from the stage of powder: cubic CeO_2_ (ceria, ICDD 00-081-0792), which is maintained up to 1200 °C. For the lower temperature (1000 °C), it is accompanied by akermanite as major silicate component and diopside as minor one. At 1200 °C, a rare earth-containing complex phase, named calcium cerium oxide silicate, was detected: hexagonal Ca_2_Ce_8_(SiO_4_)_6_O_2_ (ICDD 00-029-0320). Actually, the matching software proposed from the database the standard sheet of Ca_2_Eu_8_(SiO_4_)_6_O_2_, Eu also being a lanthanide; based on the similarity in ionic size (Ce^4+^(VIII) = 0.97 Å, Eu^3+^(VIII) = 1.066 Å), we proposed the compound with Ce instead of Eu, in which Ca and Ce are placed on equivalent sites due to the small ionic radius difference (only 13%). Sm modifies the material evolution with temperature as well, holding an ionic radius of Sm^3+^(VIII) = 1.079 Å, therefore a candidate for Ca sites. In this case, akermanite was not identified anymore as crystalline phase, but only diopside. A new crystalline compound emerged for both sintering temperatures, the only one containing P, a calcium phosphate: rhombohedral Ca_3_(PO_4_)_2_ (whitelockite, ICDD 00-009-0169). Besides this, the same type of rare earth-incorporating complex phase, called calcium samarium oxide silicate, was identified for 1000 °C: hexagonal Ca_2_Sm_8_(SiO_4_)_6_O_2_ (ICDD 00-029-0365). The latter disappears when the temperature is raised from 1000 to 1200 °C, most probably because of its instability at heating, subjected to a process of melting or decomposition; limited data are available on this compound, which means that the previous statements are mere assumptions. It can be stated that Sm favors the mass crystallization by modifying the system thermodynamics, so that to trigger the ordering of a phase including P, element that was previously distributed within the glassy phase.

It is also noteworthy mentioning that sometimes the diffraction peaks are shifted compared to the standard positions, which indicates a certain degree of doping of the lattices; this could be precisely assessed only by advanced techniques of characterization, such as X-ray photoelectron spectroscopy, together with the proportion of vitreous phase. Moreover, such materials embedding residual amorphous domains are desirable compared to fully crystallized candidates due to the ease of attack, starting from the weak points found in glass, within environments that simulate the physiological conditions.

In brief, Sm guarantees the most distinct behavior from the mineralogical point of view, while the associated scaffolds could be the most interesting ones when employed as medical substitutes, based on the existence of calcium phosphate as crystalline compound and integration of Sm cations as dopant for the ordered networks or as weakly bound entities within the amorphous phase.

It is well known that akermanite possesses favorable mechanical properties and excellent biological performance. Indeed, for scaffolds fabricated via laser sintering, the results indicate an optimum compressive strength of about 6 MPa, good bioactivity and excellent cytocompatibility [46]. When synthesized by mechanical milling and sintering at 1200 °C, akermanite displayed similar mechanical characteristics, meaning in the range of human cancellous bone, and induced obvious apatite formation on the surface after only 7 days of soaking; thus, the corresponding ceramics might be potentially used as bone repair biomaterials for non-load bearing applications [47]. Akermanite ceramics were also prepared by thermally treating the sol-gel powder compacts at 1370 °C; the results indicated bioactivity and improved mechanical features compared with those of hydroxiapatite ceramics [48]. Akermanite-based glass ceramics were successfully obtained via sintering and crystallization of glass powder pellets at low temperatures between 750 and 800 °C; they were proven to be suitable for use in restorative dentistry, mainly starting from the aesthetic properties [49].

**Figure 8:**
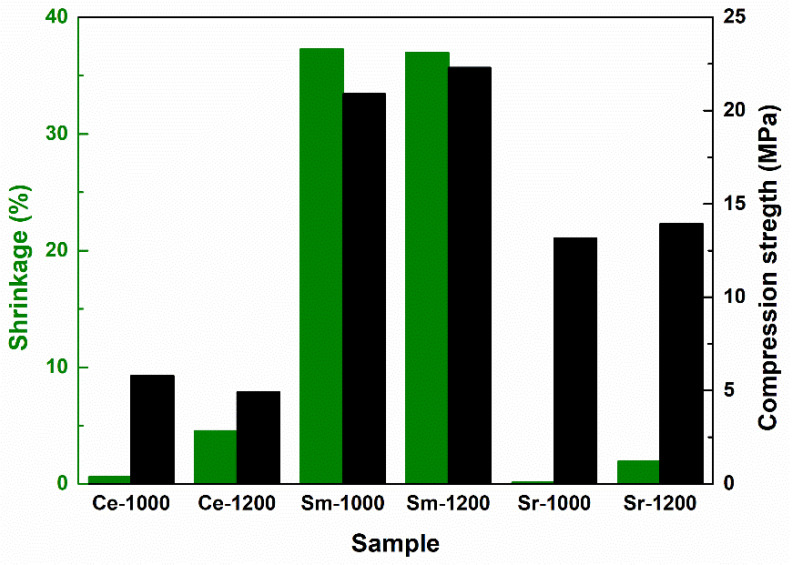
Shrinkage and compression strength for the sintered scaffolds.

Knowing that the shrinkage of the green bodies occurring during the thermal treatment is rather well correlated with the mechanical properties, this parameter was plotted together with the compression strength (Figure 8), in order to elucidate the role of material contraction and voids filling on the response under compressive loads. The best results were achieved for Sm, with values around 20 MPa, accompanied by a huge volume shrinkage (above 35%). This is not surprising, bearing in mind the great capability of Sm as dopant. Based on the mineralogical findings (Figure 7), Sm does not generate any independent phase, but is integrated within other crystalline compounds, modifying their structure through the deformations and tensions locally engendered, ultimately beneficial for the mass transfer through diffusion. In other words, the corresponding scaffolds are mechanically superior based on a lower proportion of porosity and more extended intergranular bridges (Figure 3b,c), as well as a higher degree of crystallinity (Figure 7). On the other hand, Sr addition results in a compression strength reaching 13 MPa, together with recording contractions of maximum 2%; this behavior proves once again the significant contribution of Sr to ensuring a solid resistance structure. Finally, the incorporation of Ce involves values of 5–6 MPa, whilst keeping the shrinkage under 5%. Considering the effect of the sintering temperature increase from 1000 to 1200 °C, in the case of Sm and Sr, the two parameters (shrinkage and compression strength) exhibit a slight gain, as an expected trend for a ceramic body. However, the situation is different when it comes to Ce, for which the compression strength registers a decrease of approximately 1 MPa, probably due to an advanced crystallization of CeO_2_ domains at high temperature, process that could lead to structure tensioning within the framework of material shrinkage through intense diffusion and material repositioning. Summarizing, all values found for the compression strength are more than desirable for hard tissue applications, proving the potential of the designed scaffolds in regenerative medicine. Typical values are from several MPa up to hundreds of MPa, depending on the type of bone [50].

When designing new bone substituting materials, many factors must be considered, from pore size and shape, porosity degree and interconnectivity to surface chemistry and mechanical stability [51]. Thus, a delicate balance occurs between the necessity of allowing cells migration and proliferation, as well as vascularization, and the obligation to ensure the structural integrity through adequate mechanical properties [52]. Indeed, the existence of a porous surface creates the premises of improved bioactivity and interlocking between the implant biomaterial and surrounding natural tissue, with beneficial implications on the interface stability; although increased porosity and pore size favor bone ingrowth and enable permeability, these compromise the mechanical strength, which can be critical in the case of load-bearing bones [53]. Concluding, by optimizing scaffolds morphology, namely pore size and material density, the proposed compositions could represent important candidates in the field, since the attained mechanical features are appropriate.

Goel et al. [54] investigated the influence of SrO on the sintering behavior, crystalline structure and surface reactivity of melt-quenched bioactive glasses with similar composition; they concluded that Sr embedding renders dense and mechanically resistant glass ceramics, retards the hydroxyapatite-forming ability and significantly decreases the degradation in physiological fluids. Kaur et al. [55] studied the influence of CeO_2_ on the structure, degradation, bioactivity, osteoblasts proliferation, oxidative stress and cell apoptosis induction in sol-gel-derived bioceramics with similar composition; they reported that Ce addition decreases the degradation rate, delays hydroxyapatite growth and improves the antioxidant properties.

**Figure 9:**
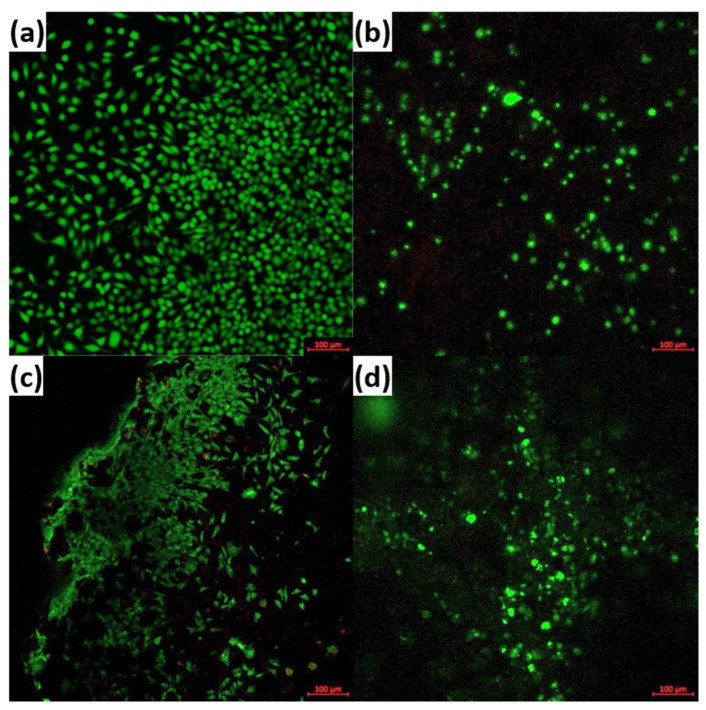
Representative fluorescence images for the synthesized scaffolds in contact with fibroblast cells for 7 days: (**a**) Control, (**b**) Ce-1000, (**c**) Sm-1000 and (**d**) Sr-1000.

Since the differences in terms of compressive strength are not so important when the thermal treatment temperature is regarded and it is not desirable to have un unjustified energy consumption in material preparation, the scaffolds sintered at 1000 °C were selected for a preliminary assessment of the biological response when in contact with fibroblast cells for 7 days. Thus, the confocal microscopy images in Figure 9 show the viability of the tested cell line after fluorescent marking of living cells in green and dead cells in red. As it can be observed, the number of living entities is predominant for all approached cations (Ce^4+^, Sm^3+^, Sr^2+^). Analyzing comparatively the situation displayed for Control (cells grown on glass slides in similar conditions) and the three scaffolds, it can be stated that the greatest adhesion and the most favored metabolism can be found in the case of Sm, for which the individual cells spread extensions and build networks, in a strong correlation with the surface provided by the sample; the cellular layout seems to reproduce the microstructure of the porous support after penetrating the available empty spaces between the constituent blocks. Ce and Sr-incorporating samples are populated by fibroblasts to a lower extent, the investigated cells preserve their rounded shape, but the image associated with Sr-incorporating system indicates a depth disposing of the cellular culture through different shades of green, more or less intense. Even for these two cases, it is possible to attain a better evolution at longer testing intervals. Overall, the LIVE/DEAD assay confirms the beneficial influence of Sm on the cells development in simulating conditions, as it was also reported by other researchers [30,31,32,33,34,35].

## 4. Conclusions

Ceramic scaffolds were designed and fabricated within the oxide system SiO_2_‒P_2_O_5_‒CaO‒MgO, incorporating supplementary valuable cations (Ce^4+^, Sm^3+^, Sr^2+^) with the aim of improving both their mechanical and biological properties. Sol-gel method was applied for the preparation of the precursor powders, that were subsequently converted into scaffolds by sintering at 1000 and 1200 °C. The morphological evaluation demonstrated their porous structure and porosity reduction (visually quantified) with temperature increasing. All targeted elements were identified within the samples, as well as specific bonding and grouping ability. The phase composition was revealed as combinations between regular calcium magnesium silicates and rare earth-containing complex crystalline compounds or simple oxides. From the mechanical point of view, Sm was the best choice, followed by Sr and then Ce. Preliminary biological evaluation highlighted the positive effect of Sm cations on the metabolism of fibroblast cells after 7 days of culturing. Such complex mineral scaffolds containing therapeutic ions are regarded as potential substitutes for bone and dental replacement applications.

## Figures and Tables

**Table 1:** Oxide compositions of the developed materials.

Code	SiO_2_	P_2_O_5_	CaO	MgO	CeO_2_	Sm_2_O_3_	SrO
	*(mol%)*						
**Ce**	38	4	36	18	**4**	0	0
**Sm**					0	**4**	0
**Sr**					0	0	**4**

**Table 2:** Elemental composition of the developed materials.

Sample	Si	P	Ca	Mg	Ce/Sm/Sr	O
**Ce**	32.39	6.12	24.42	10.61	11.48	14.97
**Ce-1000**	23.82	3.95	34.27	10.36	13.34	14.26
**Ce-1200**	27.98	3.88	29.19	11.03	9.15	18.77
**Sm**	27.25	17.89	13.82	3.60	24.51	12.92
**Sm-1000**	25.46	9.15	26.8	9.10	6.92	22.57
**Sm-1200**	23.03	9.90	36.54	7.29	9.85	13.39
**Sr**	32.16	4.77	35.39	7.88	5.77	14.03
**Sr-1000**	20.00	4.13	38.00	9.04	11.41	17.43
**Sr-1200**	18.26	3.71	48.15	7.39	11.04	11.45

## Data Availability

Data sharing not applicable.
